# Supplementary material for: Mechanically induced topological transition of spectrin regulates its distribution in the mammalian cell cortex
Source: Nat Commun. 2024 Jul 8;15:5711. doi: 10.1038/s41467-024-49906-6 (PMC11231315; doi:10.1038/s41467-024-49906-6)
Supplement: Supplementary file 1 — Supplementary Information [file 41467_2024_49906_MOESM1_ESM.pdf]

Mechanically induced topological transition of spectrin regulates its distribution in the mammalian cell cortex

Andrea Ghisleni[1], Mayte Bonilla-Quintana[2], Michele Crestani[1&], Zeno Lavagnino[1], Camilla Galli[1$], Padmini Rangamani[2*], Nils Gauthier[1*]

[1]IFOM ETS, The AIRC Institute of Molecular Oncology, Via Adamello 16, 20139, Milan, Italy
[2]Department of Mechanical and Aerospace Engineering, University of California San Diego, La Jolla CA 92093, USA

[&]Laboratory of Applied Mechanobiology, Department for Health Sciences and Technology, ETH Zürich, CH-8092 Zürich, Switzerland
[$]Humanitas Cardio Center, IRCCS Humanitas Research Hospital, Via Manzoni 56, 20089, Rozzano (Milan), Italy

* corresponding authors: nils.gauthier@ifom.eu ; prangamani@ucsd.edu

Supplementary Information

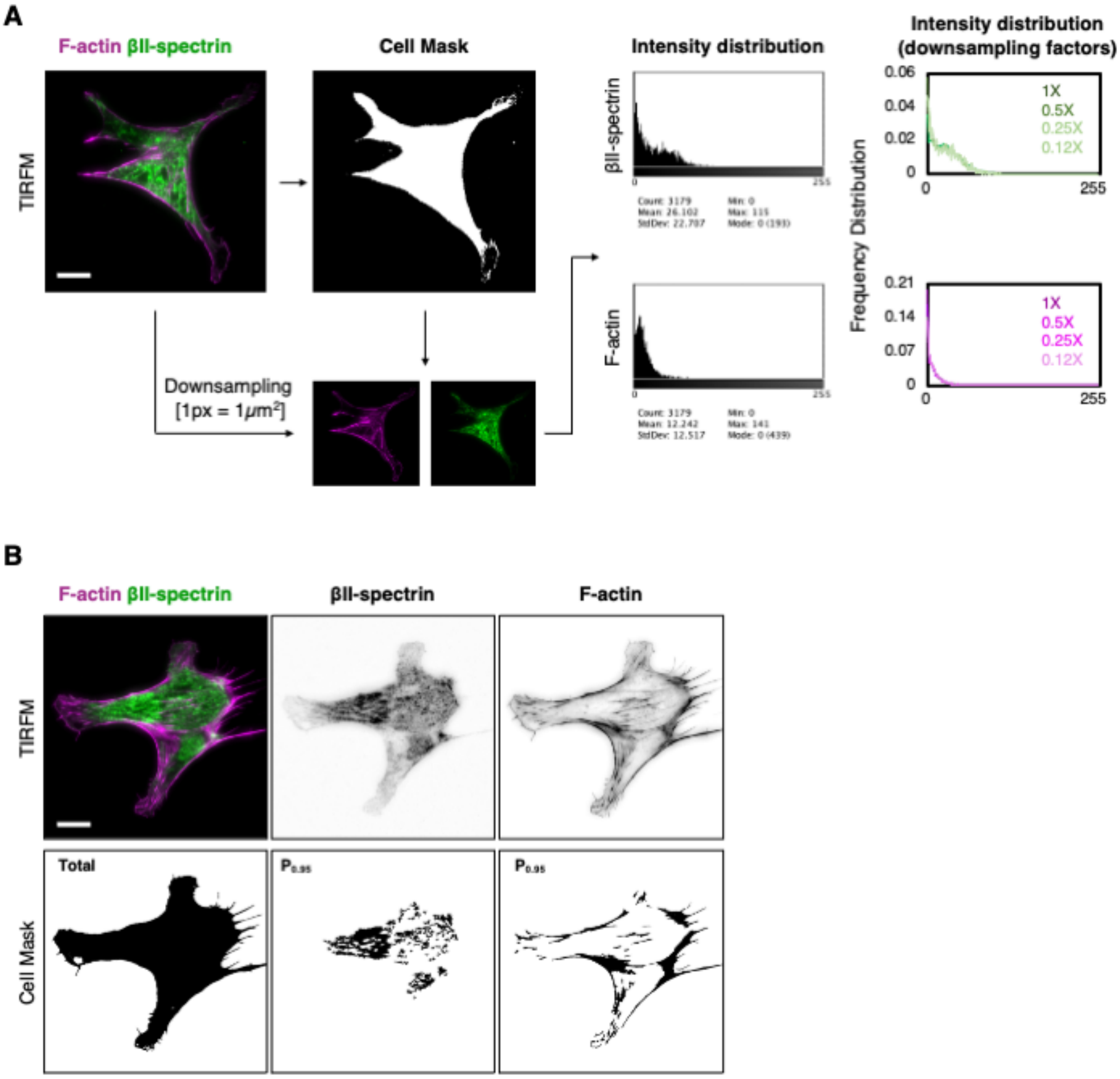

Supplementary Figure 1 A) Experimental pipeline implemented to analyze the distribution of fluorescence intensities shown in Figure 1B. Background-subtracted TIRFM images (50 pixels radius, scale bar 20 μm) were used to generate a binary cell mask; single channel images were downsampled to 1 pixel = 1 μm^2, intensities were averaged during the rescaling. Intensity distributions for each single channel image was obtained within the cell mask, with the total pixel count matching the cell area in μm^2. Frequency distributions for n=40 cells were merged and shown in Figure 1D. Fiji histogram output related to single-cell intensity distribution is shown. Different downsampling factors were applied on the cell shownto exclude biases. B) Representative analysis of $P_{0.95}$ signal intensity. Background-subtracted (50 pixels radius) and noise filtered images were thresholded by considering only pixels with the 5% most intense signal ($P_{0.95}$ of total signal intensity distribution). Particle analysis was then performed to extract area and shape descriptor parameters. Representative mask images for βII-spectrin (green) and F-actin (phalloidin magenta) are shown, with corresponding $P_{0.95}$ masks (scale bar 20 μm).

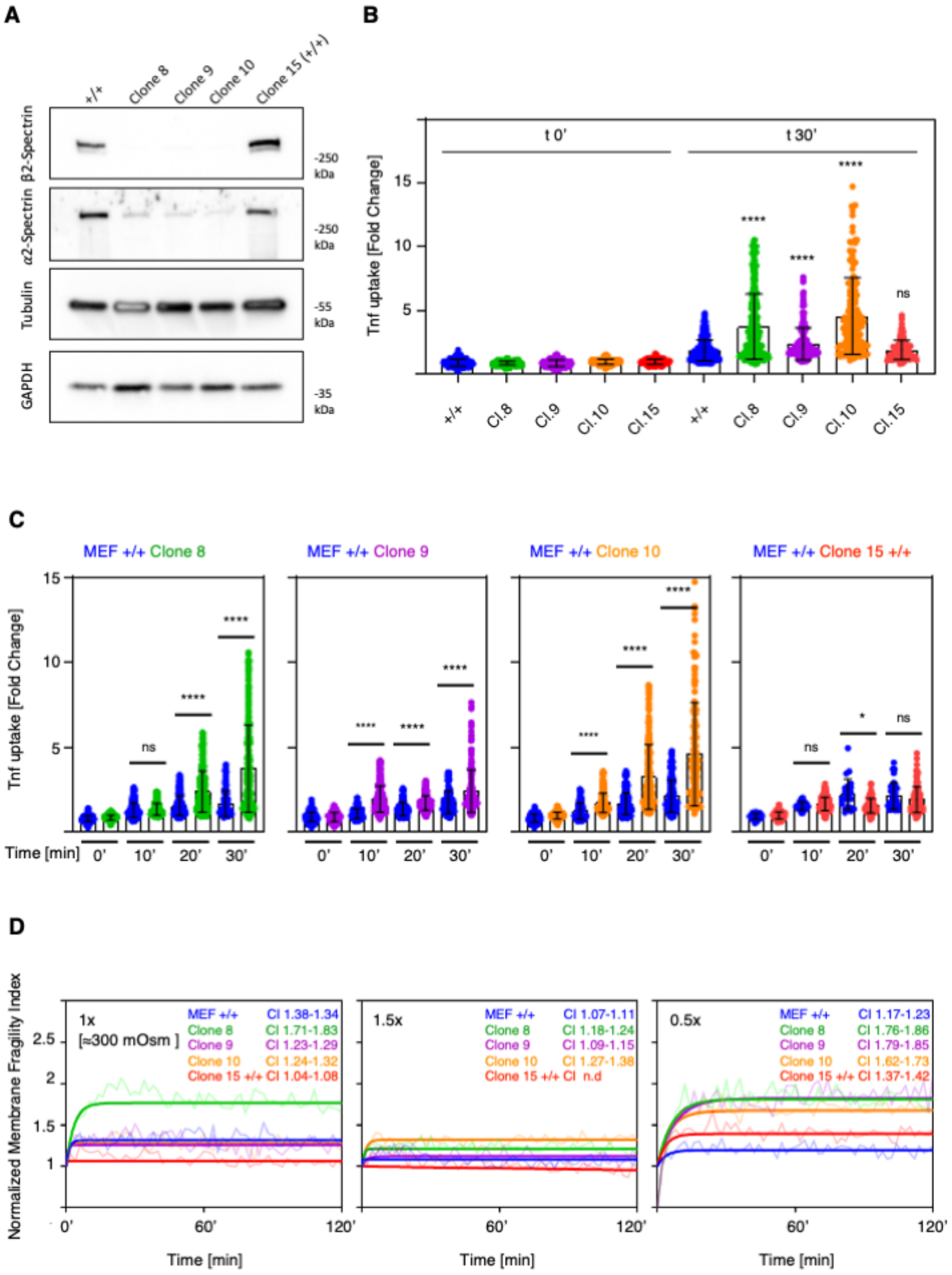

A
+/+
Clone 8
Clone 9
Clone 10
Clone 15 (+/+)
β2-Spectrin
α2-Spectrin
Tubulin
GAPDH
-250 kDa
-250 kDa
-55 kDa
-35 kDa
B
t 0'
t 30'
Tnf uptake [Fold Change]
****
****
****
ns
+/+
Cl.8
Cl.9
Cl.10
Cl.15
C
MEF +/+ Clone 8
MEF +/+ Clone 9
MEF +/+ Clone 10
MEF +/+ Clone 15 +/+
Tnf uptake [Fold Change]
Time [min]
0'
10'
20'
30'
ns
*
D
Normalized Membrane Fragility Index
1x
[≈300 mOsm]
MEF +/+ CI 1.38-1.34
Clone 8 CI 1.71-1.83
Clone 9 CI 1.23-1.29
Clone 10 CI 1.24-1.32
Clone 15 +/+ CI 1.04-1.08
1.5x
MEF +/+ CI 1.07-1.11
Clone 8 CI 1.18-1.24
Clone 9 CI 1.09-1.15
Clone 10 CI 1.27-1.38
Clone 15 +/+ CI n.d
0.5x
MEF +/+ CI 1.17-1.23
Clone 8 CI 1.76-1.86
Clone 9 CI 1.79-1.85
Clone 10 CI 1.62-1.73
Clone 15 +/+ CI 1.37-1.42
Time [min]
0'
60'
120'

Supplementary Figure 2 A) Western blot analysis of different clonal populations highlighted the complete depletion of βII-spectrin in clones 8, 9, 10, but not clone 15 and parental MEF +/+. Interestingly, expression levels of αII-spectrin were severely reduced in *sptbn1* KO clones. B-C) Time course internalization of rhodamine-labelled transferrin in different clonal populations (n = 248-432(+/+), 346-253(Cl.8), 249-209(Cl.9), 224-169(Cl.10) and 125-112(Cl.15) cells in 2 independent experiments, data are presented as mean ± SD, statistical analysis one-way ANOVA with multiple comparisons, **** p value < 0.0001). Results are presented at t 0' (negative control) and the end point of the experiments (t 30') in B, and at different time steps for each clonal population compared to MEF +/+ independently in C (n > 100 cells in 2 independent experiments, statistical analysis one-way ANOVA with multiple comparisons, * p value < 0.05, **** p value < 0.0001). Membrane Fragility was measured by exposing different clonal populations to Ringer's buffers with different fractional osmolarity. Given the noisy nature of the kinetic analyses, results are presented by fitting the curves with one-phase exponential equation to obtain the Normalized Fragility Indexes (corresponding to the plateau of the fitting, see Supplementary Table 2) and the confidence intervals ($CI_{0.95\%}$). Overall, *sptbn1* KO MEFs displayed increased fragility upon exposure to 0.5x hypotonic media. Interestingly *sptbn1* KO Clone 8 displayed increased fragility also in isotonic conditions (n=6 independent experiments).

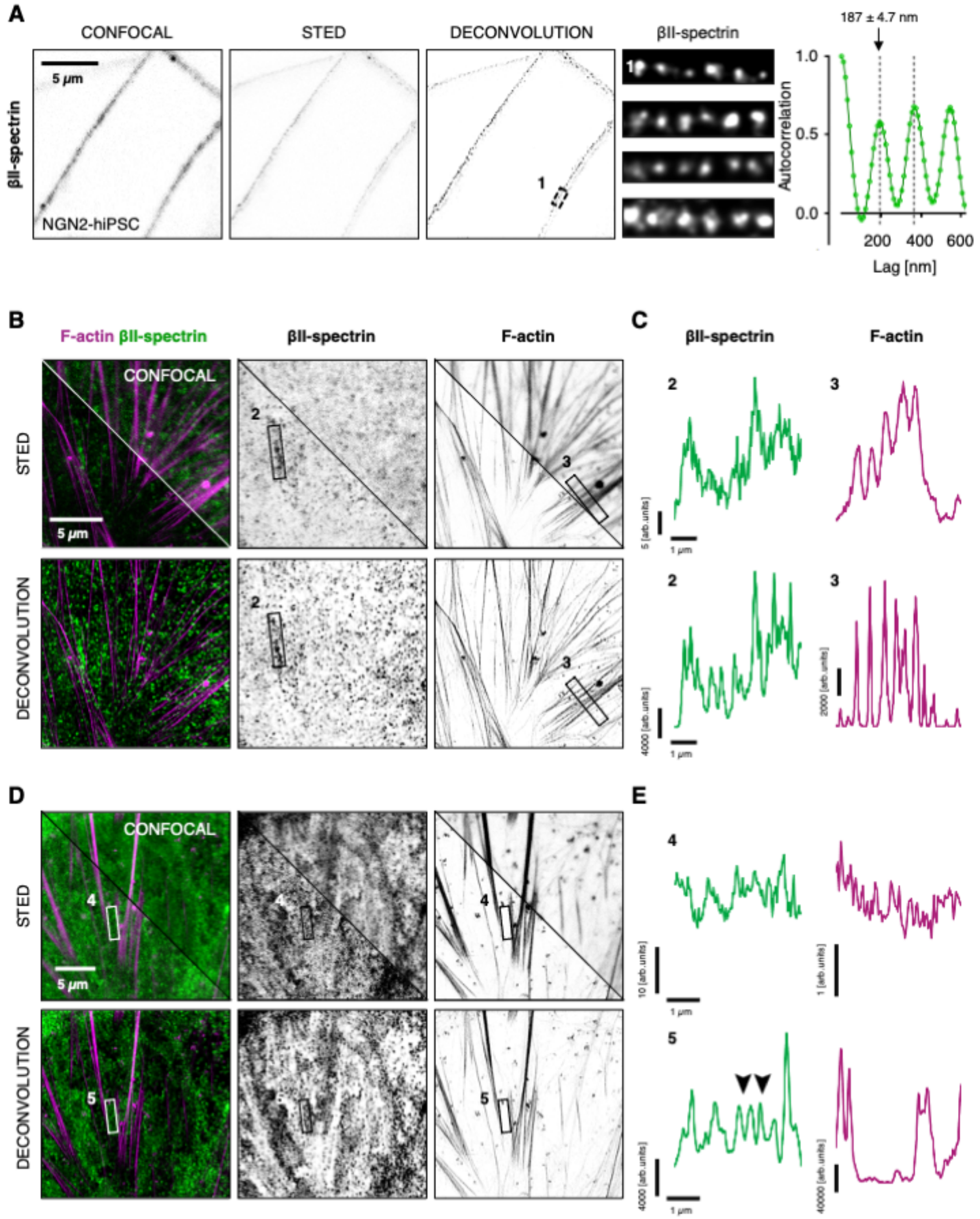
A
CONFOCAL
STED
DECONVOLUTION
βII-spectrin
βII-spectrin
5 μm
NGN2-hiPSC
1
187 ± 4.7 nm
Autocorrelation
Lag [nm]
B
F-actin βII-spectrin
βII-spectrin
F-actin
CONFOCAL
STED
DECONVOLUTION
5 μm
C
βII-spectrin
F-actin
2
3
D
E
4
5
1 μm

Supplementary Figure 3 A) Representative Confocal and STED images of NGN2-hiPSC cortical neurons at day 18 of differentiation, immunolabelled for βII-spectrin. When individual axons were observed, 1-1.5 μm long segments were analyzed for epitope spacing by 2D autocorrelation. Average peak-to-peak distance resulted 187 ± 4.7 nm (n = 5). B) Representative Confocal and dual-color STED images of MEFs immunolabelled for βII-spectrin (green) and phalloidin (F-actin, magenta) are shown. The same images are presented after deconvolution. C) Line scan analysis of signal intensities, corresponding to the black boxes (2 and 3), are reported and highlighted the improvement in resolution for F-actin (3). Spectrin signal in the ventral side of the cell, showed mild improvement in resolution between confocal and raw STED images (2). After deconvolution, clusters were analyzed but failed to show substantial improvement compared to confocal analysis or the F-actin counterpart. D-E) Only occasional periodicity (arrowheads) in the βII-spectrin channel after deconvolution could be observed at regions confined between stress fibers (4-5, white and black boxes). All images are representative of at least 3 independent experiments.

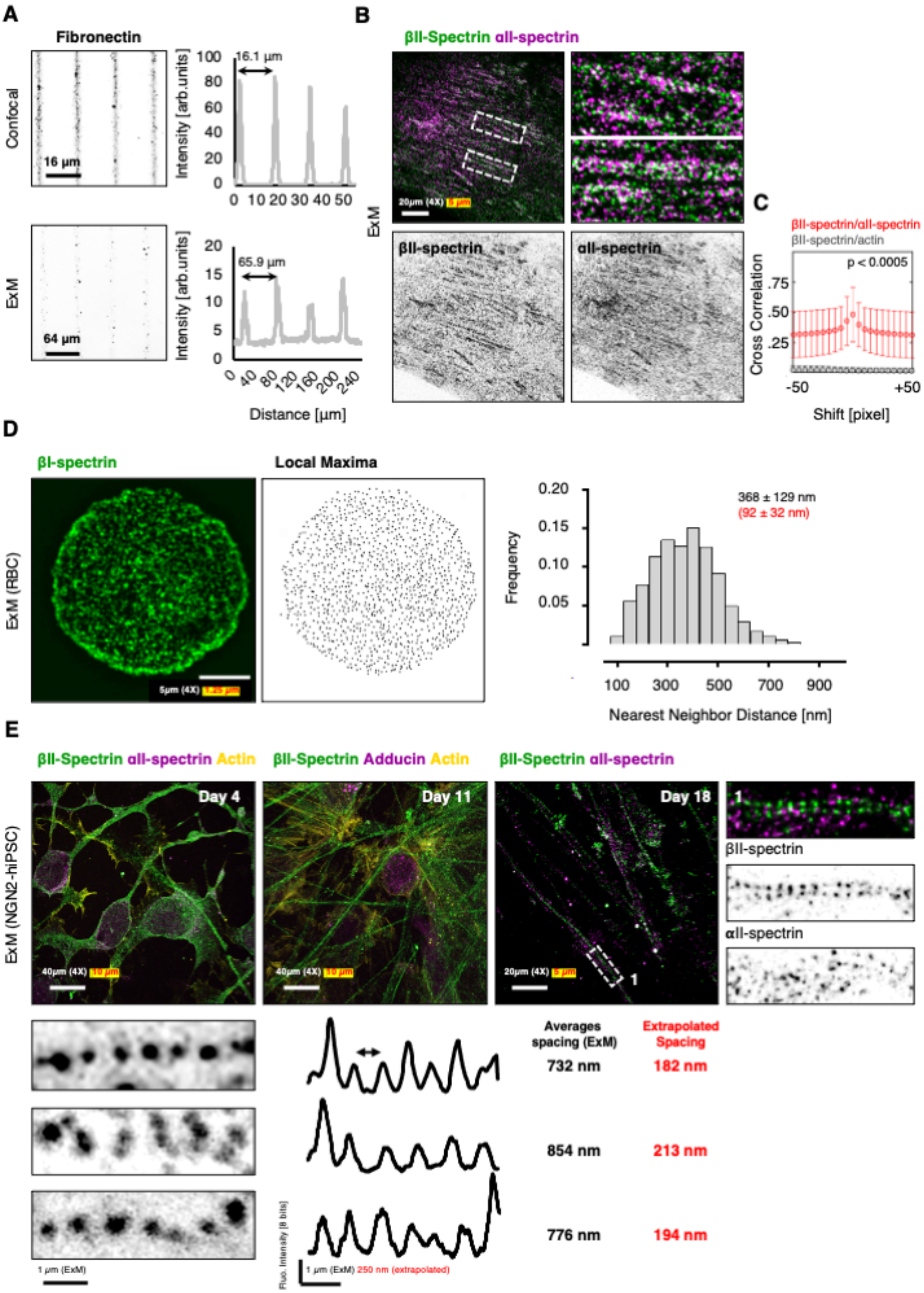

A
Fibronectin
Confocal
16 µm
16.1 µm
Intensity [arb.units]
ExM
64 µm
65.9 µm
Distance [µm]
B
βII-Spectrin αII-spectrin
ExM
βII-spectrin
αII-spectrin
C
βII-spectrin/αII-spectrin
βII-spectrin/actin
p < 0.0005
Cross Correlation
Shift [pixel]
D
βI-spectrin
Local Maxima
ExM (RBC)
368 ± 129 nm
(92 ± 32 nm)
Frequency
Nearest Neighbor Distance [nm]
E
βII-Spectrin αII-spectrin Actin
βII-Spectrin Adducin Actin
βII-Spectrin αII-spectrin
Day 4
Day 11
Day 18
ExM (NGN2-hiPSC)
βII-spectrin
αII-spectrin
Averages spacing (ExM)
Extrapolated Spacing
732 nm
182 nm
854 nm
213 nm
776 nm
194 nm
1 µm (ExM)
1 µm (ExM) 250 nm (extrapolated)

Supplementary Figure 4 A) The estimate expansion factor for the ExM protocol was obtained by immunostaining fibronectin-coated microprinted lines with an anti-fibronectin antibody, and imaging was performed before and after expansion by confocal microscopy. The photolithography mask is designed with continuous lines of 4 μm thickness and 12 μm gaps. The profile plots are showing the expected 16.1 μm peak-to-peak distance (12+4 μm), and the resulting 65.9 μm after gelation and expansion: $\approx$4x expansion factor is achieved. B) Representative ExM image of MEFs immunolabelled for βII-spectrin (green) and αII-spectrin (magenta) is shown (scale bar 20 μm). Zooms (white dashed boxes) are shown to highlight the intermingled nature of the two epitopes. C) 2D cross correlation analysis of dual immunolabelled MEFs imaged by ExM (window size 50 x 50 pixels): βII-spectrin/αII-spectrin (red) and βII-spectrin/β-Actin (grey, data are presented as mean ± SD, n= 7-9 cells, statistical analysis two-way ANOVA). D) ExM of murine RBCs immunolabelled for βI-spectrin (green) was performed. Local Maxima analysis and Nearest Neighbor Distance distributions was calculated to report the inter-epitope distance (368 ± 129 nm, 92 ± 32 nm when corrected for the 4x expansion factor, n= 8 cells). E) ExM of NGN2-hiPSC cortical neurons at different time points during differentiation, immunolabelled for βII-spectrin (green), αII-spectrin or adducin (magenta) and ExM-compatible phalloidin (yellow). The expected periodic pattern for βII-spectrin was identified and highlighted in zoom 1. Line scan analysis reported the expected βII-spectrin epitope inter-distance in neuronal axons for three independent neuronal patches. The periodic pattern was not identified for the other stainings. All images are representative of at least 3 independent experiments.

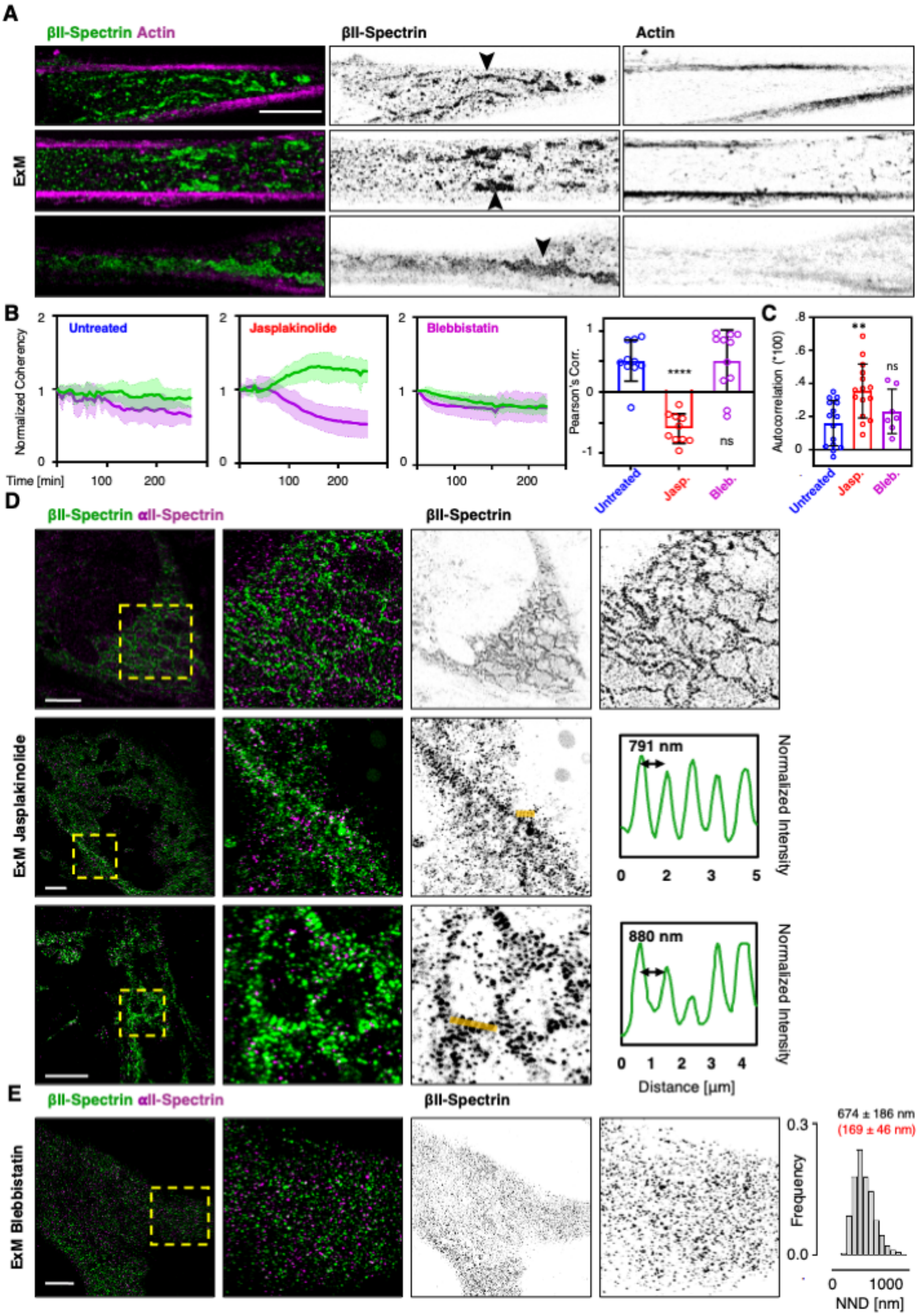
A
βII-Spectrin Actin
βII-Spectrin
Actin
ExM
B
Untreated
Jasplakinolide
Blebbistatin
Normalized Coherency
Time [min]
100
200
Pearson's Corr.
****
ns
Untreated
Jasp.
Bleb.
C
Autocorrelation (*100)
**
ns
D
βII-Spectrin αII-Spectrin
βII-Spectrin
ExM Jasplakinolide
791 nm
880 nm
Normalized Intensity
Distance [µm]
E
ExM Blebbistatin
674 ± 186 nm
(169 ± 46 nm)
Frequency
NND [nm]

Supplementary Figure 5 A) More representative ExM images of MEFs seeded on adherent microprinted pattern and immunolabeled for endogenous βII-spectrin (green) and actin (magenta). Black arrowheads indicate regions of spectrin accumulation without periodic organization (qualitative evaluation). B) Normalized coherency over time between the two fluorescent channels is reported for untreated cells (n = 10 cells), Jasplakinolide and Blebbistatin treatments (n = 11 cells). Pearson's correlation indexes between Spectrin and Actin signals over the time series are reported (data are presented as mean ± SD, statistical analysis: one-way ANOVA with multiple comparisons, **** p value <0.0001). C) Auto-correlation coefficients of βII-spectrin signals in the field of view of ExM images are plotted for each treatment (n=15 independent images for Untreated and Jasplakinolide, n=7 independent images for Blebbistatin; data are presented as mean ± SD, statistical analysis one-way ANOVA with multiple comparisons, ** p< 0.005). Additional representative images are shown for Jasplakinolide (D) and Blebbistatin (E) treated MEFs (scale bars = 20 μm). Zooms are highlighted by the dashed yellow boxes. Intensity line-scans across the yellow rectangles are shown in the graphs and refer to the effect of Jasplakinolide treatment. Nearest Neighbour Distance distribution of the βII-spectrin under Blebbistatin treatment is shown in E. All images are representative of at least 3 independent experiments.

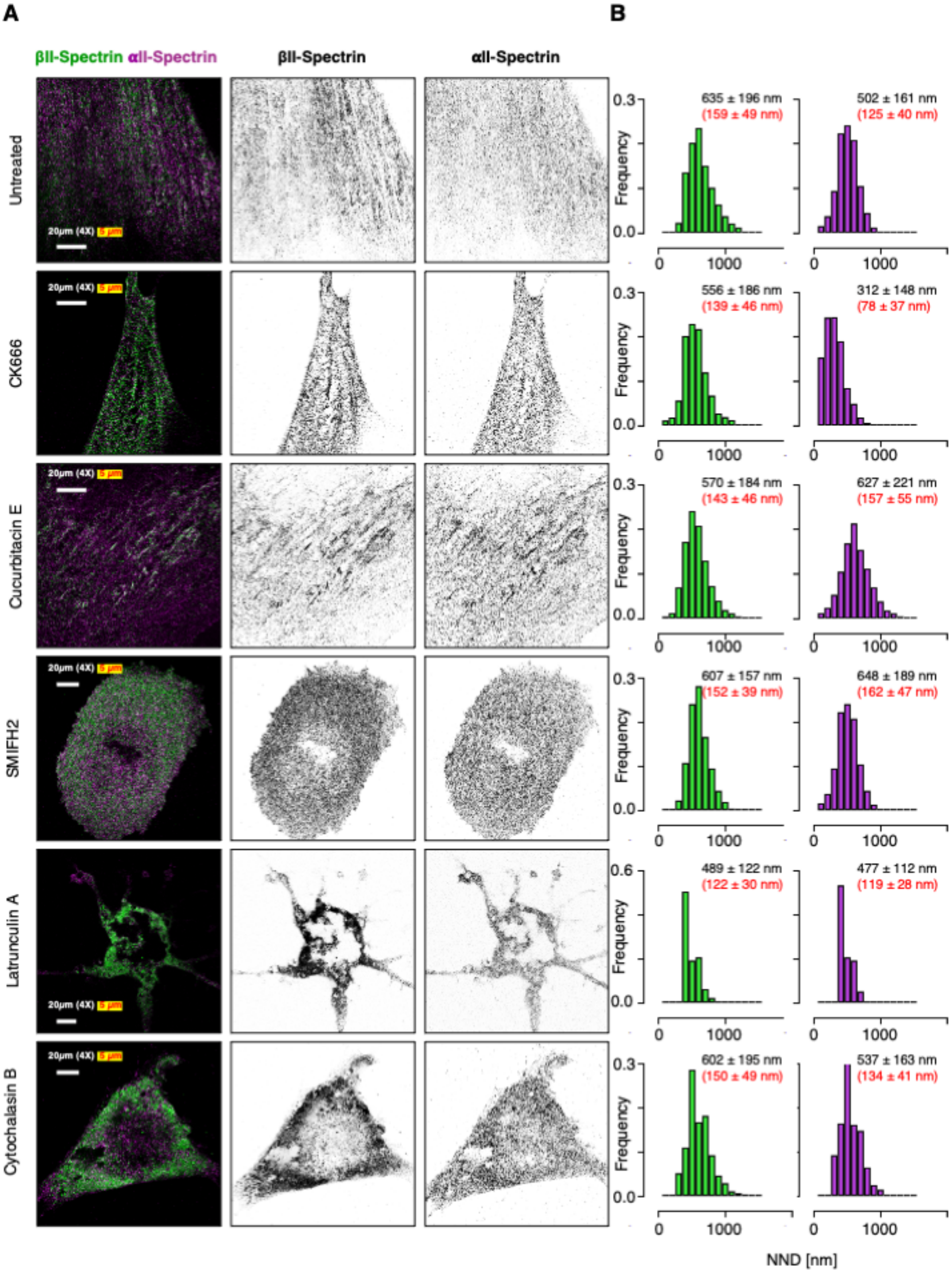

A
B
βII-Spectrin αII-Spectrin
βII-Spectrin
αII-Spectrin
Untreated
CK666
Cucurbitacin E
SMIFH2
Latrunculin A
Cytochalasin B
20μm (4X)
5 μm
Frequency
0.3
0.0
0.6
0
1000
635 ± 196 nm
(159 ± 49 nm)
502 ± 161 nm
(125 ± 40 nm)
556 ± 186 nm
(139 ± 46 nm)
312 ± 148 nm
(78 ± 37 nm)
570 ± 184 nm
(143 ± 46 nm)
627 ± 221 nm
(157 ± 55 nm)
607 ± 157 nm
(152 ± 39 nm)
648 ± 189 nm
(162 ± 47 nm)
489 ± 122 nm
(122 ± 30 nm)
477 ± 112 nm
(119 ± 28 nm)
602 ± 195 nm
(150 ± 49 nm)
537 ± 163 nm
(134 ± 41 nm)
NND [nm]

Supplementary Figure 6 A) ExM images of MEFs immunolabelled for endogenous βII-spectrin (green) and αII-spectrin (magenta) after treatment with different cytoskeleton-targeting compounds. Specifically, MEFs have been treated for 3-4 hours with CK666 (100 μM), Cucurbitacin E (5 nM), SMIFH2 (20 μM), Latrunculin A (1 μM), Cytochalasin B (1 μM) independently, for qualitative assessment of the global effect on the spectrin meshwork. B) Frequency distributions of the Nearest Neighbor Distances are calculated independently for the two epitopes (βII-spectrin in green and αII-spectrin in magenta), and average values are reported in the "real" scale and extrapolated from the expansion factor (red). The dual epitope mapping along the tertamer of the αII-spectrin antibody resulted in reduced NND distances in all the conditions tested. In the case of Latrunculin A treatment, NND failed to correctly discriminate between puncta due to the homogeneous clustered signal and the frequency adopted a peculiar one-tailed distribution for both βII-spectrin and αII-spectrin.
All images are representative of at least 3 independent experiments.

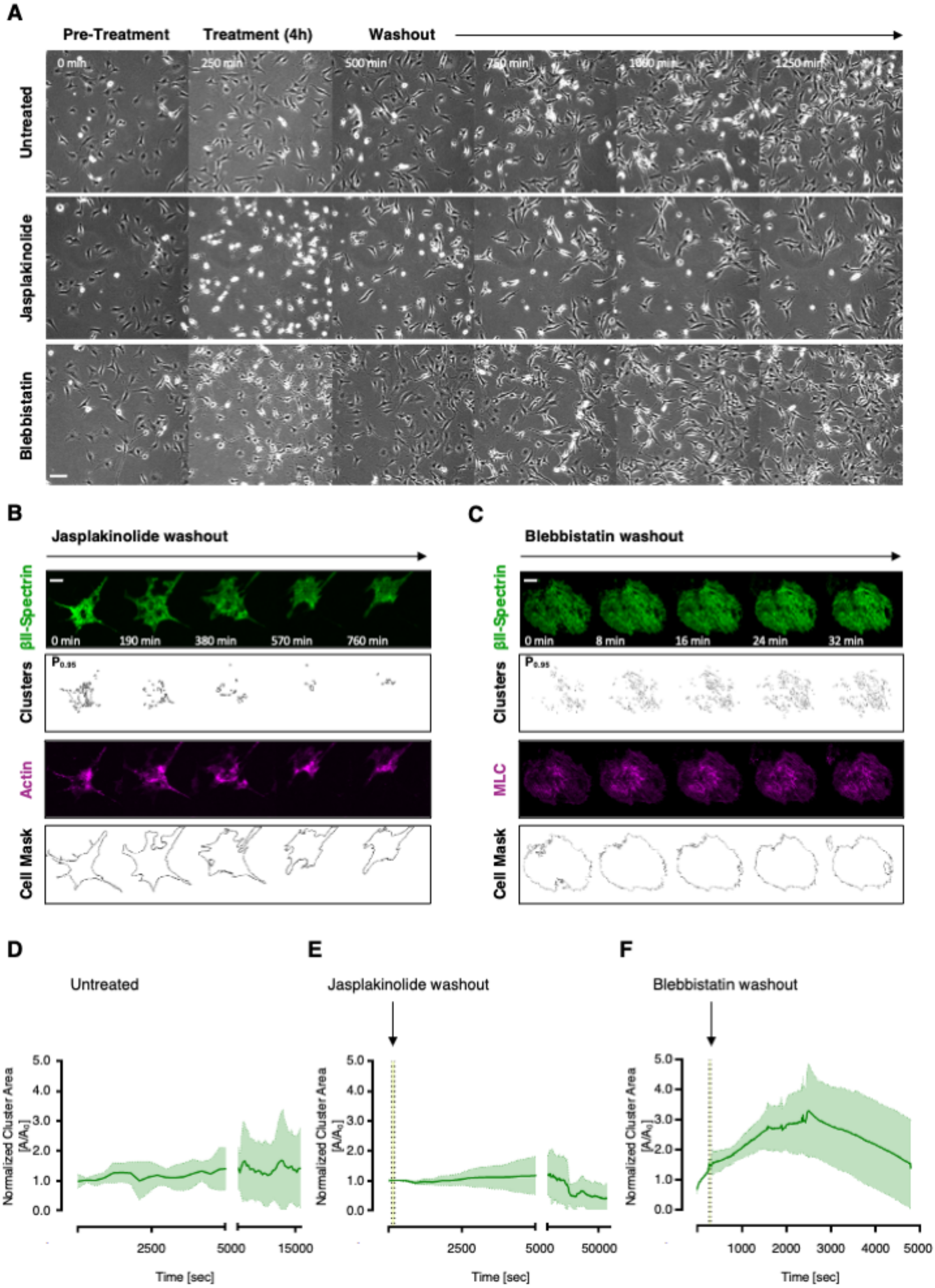
A
Pre-Treatment
Treatment (4h)
Washout
0 min
250 min
500 min
750 min
1000 min
1250 min
Untreated
Jasplakinolide
Blebbistatin
B
Jasplakinolide washout
βII-Spectrin
0 min
190 min
380 min
570 min
760 min
Clusters
$P_{0.95}$
Actin
Cell Mask
C
Blebbistatin washout
βII-Spectrin
0 min
8 min
16 min
24 min
32 min
Clusters
$P_{0.95}$
MLC
Cell Mask
D
Untreated
E
Jasplakinolide washout
F
Blebbistatin washout
Normalized Cluster Area [A/A$_0$]
Time [sec]

Supplementary Figure 7 A) Phase contrast time lapse microscopy of MEFs treated for 4 hours with Jasplakinolide 100 nM and Blebbistatin 10 μM. Cells were then followed for 16 hours after washout of the drugs to monitor the recovery of cell shape and motility (scale bare 100 μm). B-C) Live imaging by TIRF microscopy of MEFs transiently transfected with GFP-βII-spectrin (green) and RFP-actin (magenta), treated with Jasplakinolide 100 nM or Blebbistatin 10 μM. After 3-4 hours of treatment, cells were mounted in Ringer's buffer 1x and imaged for 14 hours by TIRFM, to monitor cell recovery after the washout of the drugs performed 5 minutes after the beginning of the time lapse . Relevant frames are shown (scale bare 20 μm). All images are representative of at least 3 independent experiments. D-E-F) Cluster area normalized to the initial frames was plotted and the washout is indicated by the yellow dashed lines (n = 6-7 cells, data are presented as mean ± SD). Discontinuous time on the x axis was required given the different kinetic of reactions between the two compounds. The reduction in cluster area after Jasplakinolide washout was visible after hours (5-6 hours), while Blebbistatin washout produced a transient increase within minutes.

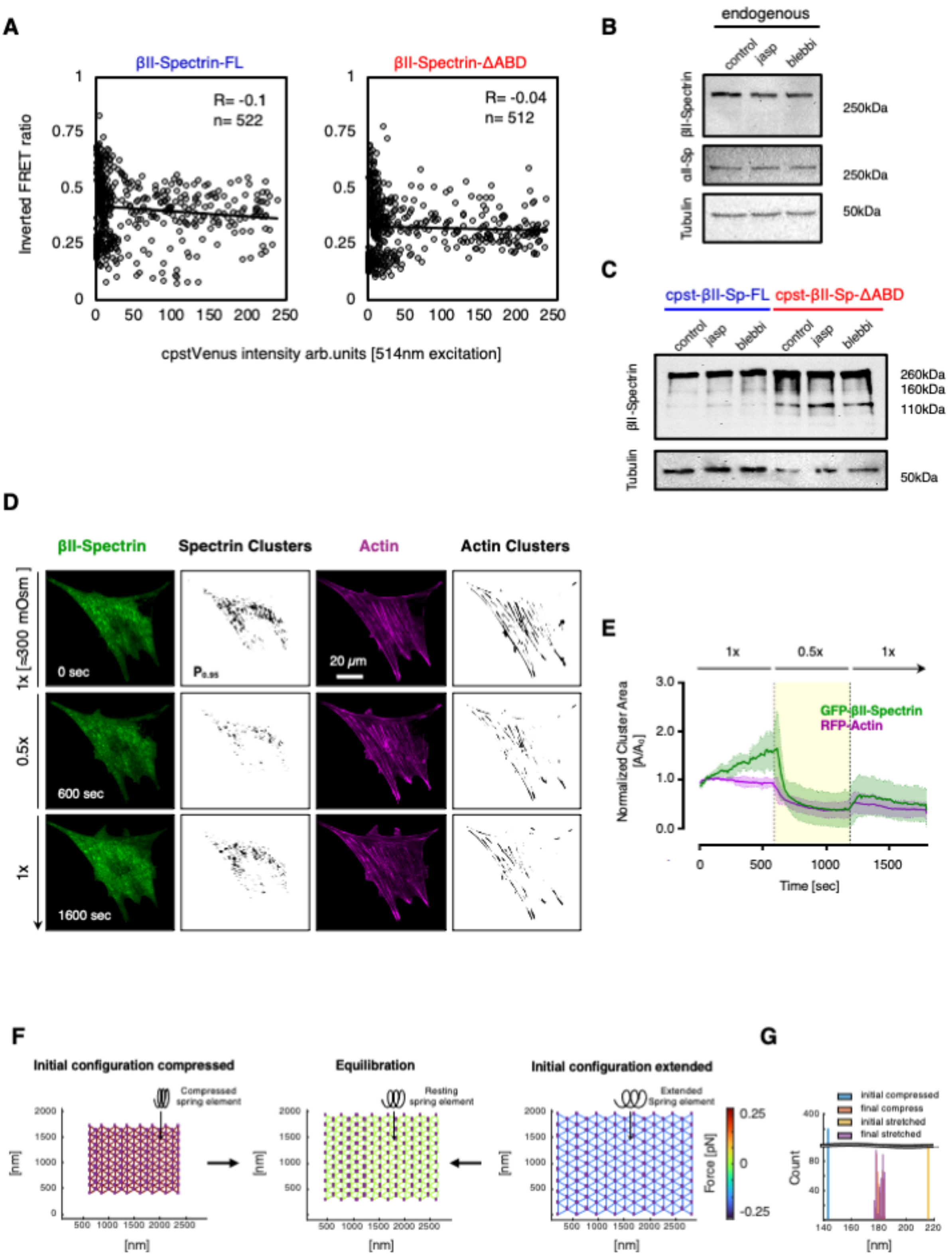
A
βII-Spectrin-FL
R= -0.1
n= 522
βII-Spectrin-ΔABD
R= -0.04
n= 512
Inverted FRET ratio
cpstVenus intensity arb.units [514nm excitation]
B
endogenous
control
jasp
blebbi
βII-Spectrin
αII-Sp
Tubulin
250kDa
250kDa
50kDa
C
cpst-βII-Sp-FL
cpst-βII-Sp-ΔABD
control
jasp
blebbi
βII-Spectrin
Tubulin
260kDa
160kDa
110kDa
50kDa
D
βII-Spectrin
Spectrin Clusters
Actin
Actin Clusters
1x [≈300 mOsm]
0.5x
1x
0 sec
600 sec
1600 sec
20 μm
E
1x
0.5x
1x
GFP-βII-Spectrin
RFP-Actin
Normalized Cluster Area [A/A₀]
Time [sec]
F
Initial configuration compressed
Compressed spring element
Equilibration
Resting spring element
Initial configuration extended
Extended Spring element
Force [pN]
[nm]
G
initial compressed
final compress
initial stretched
final stretched
Count
[nm]

Supplementary Figure 8 A) Mean inverted FRET ratio as a function of the mean Venus signal intensity (excitation at 514nm) per each cell analyzed in this study (n= 522, 512) are presented. Pearson's correlation coefficients suggests no correlation exist between total fluorescent signal and the resulting FRET ratio. B-C) Western blot analysis of total cell lysates after 3-4 hours treatment with cytoskeletal impairing drugs implemented in this study. Immunoblots with anti βII-spectrin and anti αII-spectrin antibodies exclude protein degradation and fragmentation upon treatments. Anti-Tubulin immunoblot is used as loading control. The same analysis was performed on MEFs transiently transfected with the two FRET-based constructs βII-spectrin FL and ΔABD. Drug treatments did not produce differential degradation fragments compare to the control lane. D) Fractional osmotic shocks (1x-0.5x-1x) were performed in Ringer's media, and cell reaction recorded by live TIRFM (GFP-βII-spectrin (green) and RFP-actin (magenta), scale bar 20 μm). Normalized Cluster Area of $P_{0.95}$ signal intensities were calculated for both cytoskeletal elements and plotted in E (n = 5 cells in 2 independent experiments, data are presented as mean ± SD).

F) Initial configuration of a spectrin mesh (left) where bundles are represented by edges with a spring element and short-actin filaments are represented by the mesh nodes. The length of each bundle is smaller than the resting length, acting as a compressed spring and exerting a restorative (positive) force on the mesh, depicted by the color of the edges. The configuration of the spectrin mesh in the left evolves to a relaxed state (center), in which the force generated by the spectrin bundles is minimum (equilibration). The time to reach such a state is 60 seconds. Another initial configuration of the spectrin mesh with edges larger than the resting length, acting as stretched springs (right), also relaxes to the configuration shown in the middle. G) Histogram (also reported in Figure 6C) of the initial and final length of the spectrin bundles corresponding to the meshes in (F).

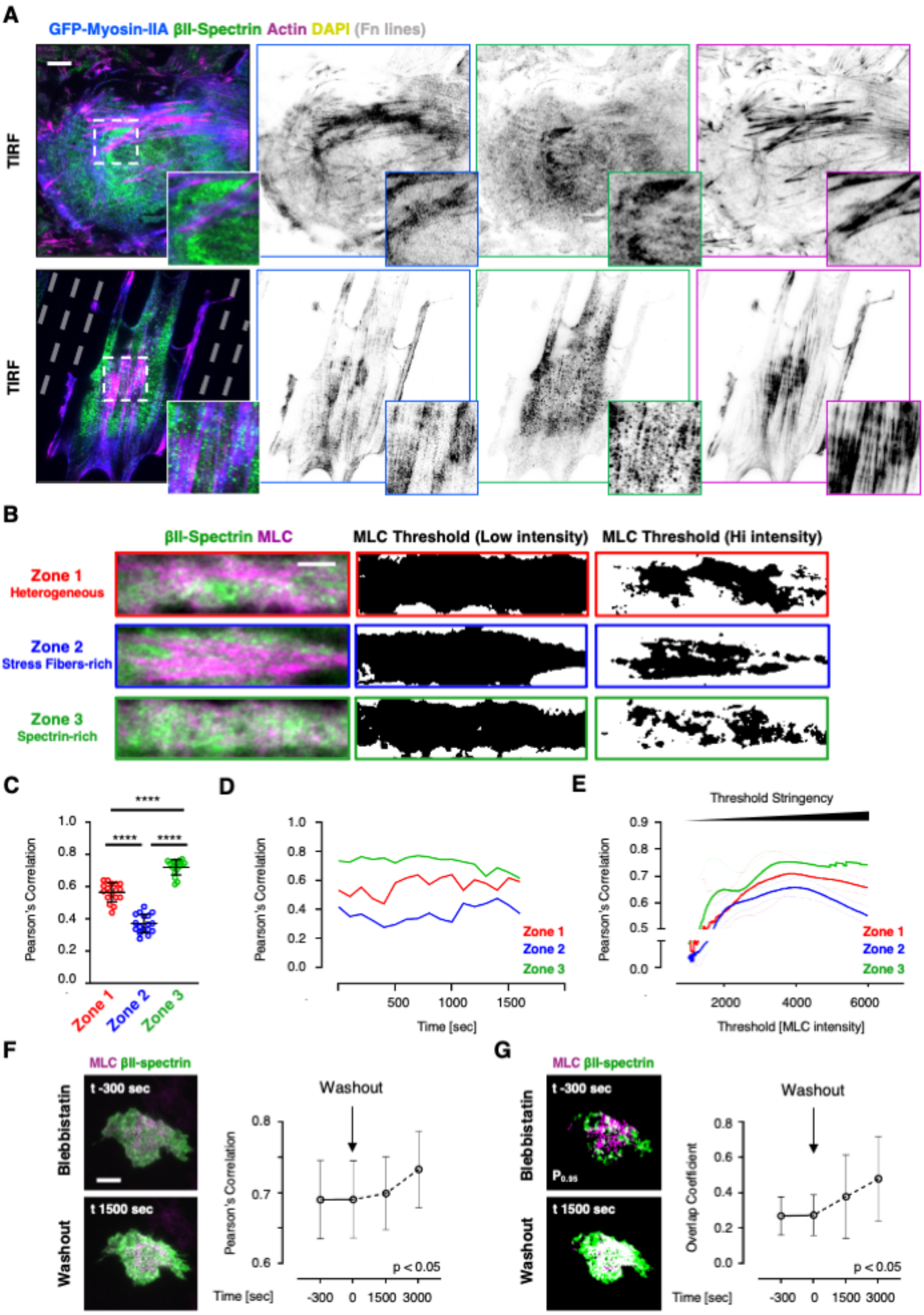
A
GFP-Myosin-IIA βII-Spectrin Actin DAPI (Fn lines)
TIRF
TIRF
B
βII-Spectrin MLC
MLC Threshold (Low intensity)
MLC Threshold (Hi intensity)
Zone 1
Heterogeneous
Zone 2
Stress Fibers-rich
Zone 3
Spectrin-rich
C
Pearson's Correlation
Zone 1
Zone 2
Zone 3
D
Pearson's Correlation
Time [sec]
E
Threshold Stringency
Pearson's Correlation
Threshold [MLC intensity]
F
MLC βII-spectrin
Blebbistatin
Washout
t -300 sec
t 1500 sec
Washout
Pearson's Correlation
Time [sec]
p < 0.05
G
MLC βII-spectrin
Blebbistatin
Washout
t -300 sec
t 1500 sec
Washout
Overlap Coefficient
Time [sec]
p < 0.05

Supplementary Figure 9 A) MEFs transiently transfected with GFP-Myosin-IIA (blue), immunolabeled for βII-spectrin (green) and F-actin (phalloidin magenta), imaged by TIRFM (scale bar = 20 µm). Overlay and single channel images are shown, as well as zoom related to the white dashed box. The same microscopy analysis is performed on MEFs seeded on microfabricated adhesive lines (4 µm adhesive cross-section, 12 µm non-adhesive surface) to force cell and cytoskeletal polarization. Images are representative of at least 3 independent experiments. B) Correlation analysis between βII-spectrin and MLC pulses has been performed in the three sub-cellular zone reported in Figure 7A. Two different thresholds in the mCherry-MLC have been applied: one at low intensity (1000 Arb.unit) and one at high intensity (4000 Arb.unit). The resulting Pearson's correlation coefficients over time are plotted in C (n=15 independent frames, data are presented as mean ± SD, statistical analysis one-way ANOVA with multiple comparisons, **** p value< 0.0001). Correlation coefficients color-coded for the three different zones are reported over time in D. E) Pearson's correlation coefficients in function of mCherry-MLC threshold stringency are reported for the three zones analyzed (n=15 independent frames, data are presented as mean ± SD). Interestingly, spectrin-rich zone 3 (green), maintains high correlation with high intensity myosin puncta, corresponding to myosin pulses. In stress fiber-rich (blue) and heterogeneous zones (red), the correlation with spectrin decrease at high MLC intensity. This dual behaviour supports the preferential correlation of spectrin with MLC pulses at spectrin-rich cortical domains, rather than the spectrin association with long-lived MLC puncta associated with stress fibers. F) Representative TIRFM images of MEFs transiently transfected with GFP-βII-spectrin (green) and mCherryMLC (magenta, scale bar = 20 µm) during Blebbistatin washout experiments. Correlation between the two channels is reported in the graph, the vertical arrow indicates the washout of the drugs (t=0 minute). The recovery phase is then followed for 3000 minutes (continuous line indicates the presence of the drug in the media, dashed line indicates the washout phase; n = 6 independent cells, data are presented as mean ± SD, statistical analysis one-way ANOVA). G) In the same dataset, $P_{0.95}$ clusters have been segmented for both fluorescent channels. Overlap coefficients between the two channels are reported at the relevant frames previously analyzed (n = 6 independent cells, data are presented as mean ± SD, statistical analysis one-way ANOVA), and highlight the same increase during myosin reactivation.

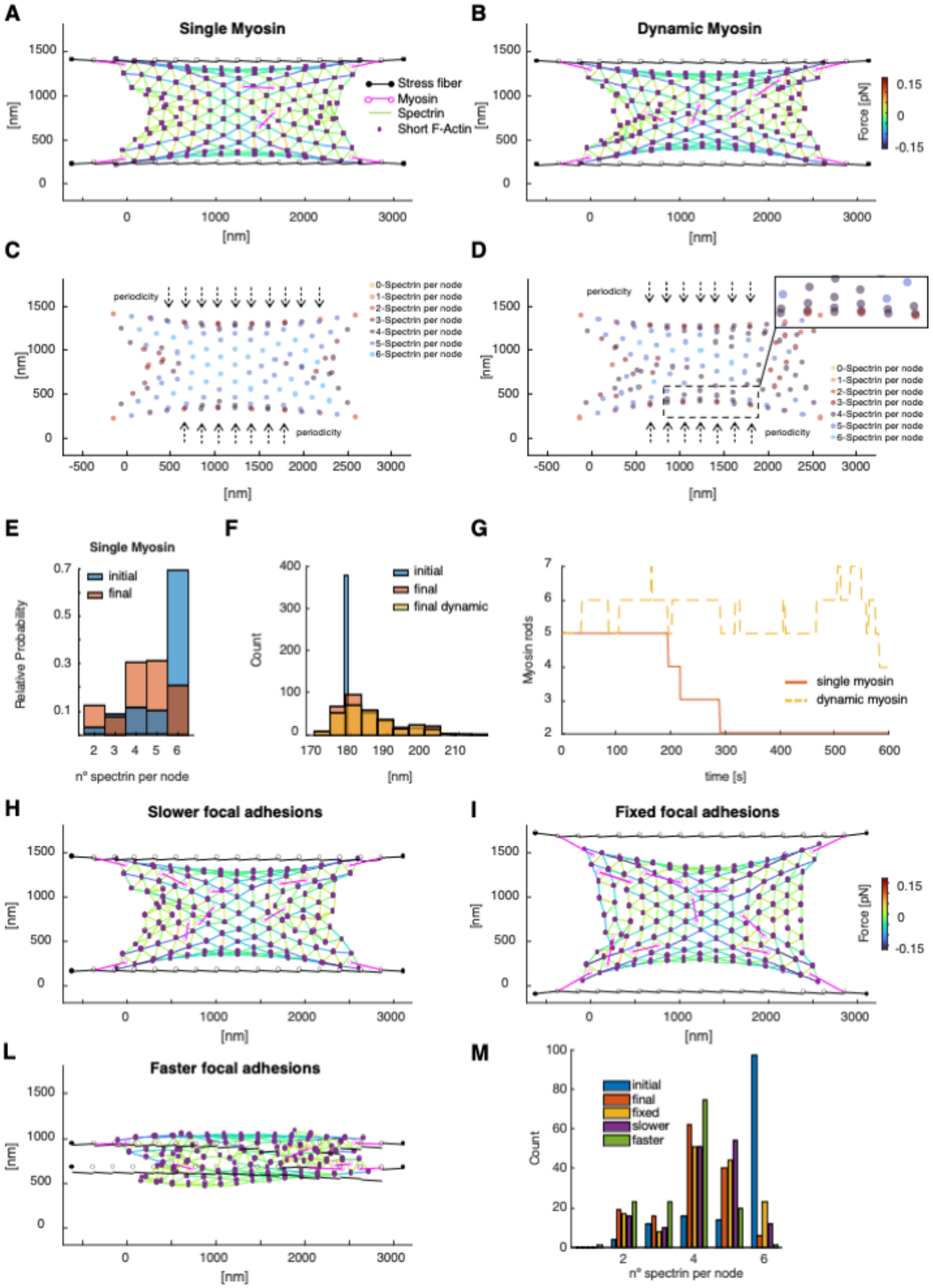
A
Single Myosin
Stress fiber
Myosin
Spectrin
Short F-Actin
B
Dynamic Myosin
Force [pN]
C
periodicity
0-Spectrin per node
1-Spectrin per node
2-Spectrin per node
3-Spectrin per node
4-Spectrin per node
5-Spectrin per node
6-Spectrin per node
D
E
Single Myosin
initial
final
Relative Probability
n° spectrin per node
F
initial
final
final dynamic
Count
G
Myosin rods
single myosin
dynamic myosin
time [s]
H
Slower focal adhesions
I
Fixed focal adhesions
L
Faster focal adhesions
M
initial
final
fixed
slower
faster
Count
n° spectrin per node
[nm]

Supplementary Figure 10 Final configuration of the network in Figure 7F for single dynamic myosin (A) and double dynamic myosin (B), color-coded for the force generated by the spring element of the spectrin bundles (Eq. 3). C-D) Same as A-B but color-coded to represent the number of attached spectrin bundles per actin node. Histograms showing the number of spectrin bundles per short actin-filaments (E) and the length of the spectrin bundles in (F). G) Evolution of the myosin rods in the spectrin network over time, corresponding to the simulations in (A-B). Final configuration of the network in Figure 7F but with the stress fibers pulled together at slower (H, vA = 0.85 nm/s) or faster (L, vA = 2.56 nm/s) velocities. I) Evolution of the same network but with fixed focal adhesions throughout the simulation. M) Histogram showing the number of connected spectrin bundles per short-actin filament for the initial (blue) and final (orange) configuration of the network in Figure 7F and the configurations in H-L. The configuration of the meshwork depends on the feedback between the spectrin meshwork and the speed with which the stress fibers can move towards one another.

Supplementary Table 1: Fluorescence Intensity distribution analysis

	Actin	βII-spectrin
Comparison of Fits		
Null hypothesis	Two phase decay	Two phase decay
Alternative hypothesis	Three phase decay	Three phase decay
P value	0.7092	<0.0001
Conclusion (alpha = 0.05)	Do not reject null hypothesis	Reject null hypothesis
Preferred model	Two phase decay	Three phase decay
F (DFn, DFd)	0.3437 (2. 10153)	11.34 (2. 8121)
Two phase decay		
Best-fit values		
Y0	0.05199	0.01859
Plateau	8.31E-02	8.64E-03
PercentFast	62.10	Unstable
KFast	0.1109	0.01956
KSlow	0.03934	0.01953
Half Life (Slow)	17.62	35.48
Half Life (Fast)	6.251	35.44
Tau (slow)	25.42	51.19
Tau (fast)	9.019	51.14
Rate constant ratio	2.818	1.001
95% CI (profile likelihood)		
Y0	0.05037 to 0.05382	0.01823 to 0.01895
Plateau	-0.0001016 to 0.0002476	-0.0001379 to 0.0001511
PercentFast	39.78 to 82.15	(Very wide)
KFast	0.08762 to 0.1582	???
KSlow	0.02647 to 0.05003	0.01882 to ???
Half Life (Slow)	13.86 to 26.18	??? to 36.84
Half Life (Fast)	4.382 to 7.910	???
Tau (slow)	19.99 to 37.77	??? to 53.14
Tau (fast)	6.322 to 11.41	???
Goodness of Fit		
Degrees of Freedom	10155	8123
R squared	0.6276	0.6336
Sum of Squares	0.3425	0.09656
Sy.x	0.005808	0.003448
AICc	-104612	-92165
Normality of Residuals		
Shapiro-Wilk (W)	0.4997	0.8620
P value	<0.0001	<0.0001

Passed normality test (alpha=0.05)?	No	No
P value summary	****	****
Constraints		
PercentFast	0 < PercentFast < 100	0 < PercentFast < 100
KSlow	KSlow > 0	KSlow > 0
Three phase decay		
Best-fit values		
Y0	0.05232	0.01825
Plateau	1.95E-02	0.001908
PercentFast	8.009	65.96
KFast	0.02281	0.01457
PercentSlow	53.82	Unstable
KSlow	0.05843	0.01475
Kmedium	0.1405	0.006485
Half Life (Slow)	11.86	46.98
Half Life (Fast)	30.39	47.58
Half Life (Medium)	4.933	106.9
95% CI (profile likelihood)		
Y0	0.05051 to 0.05449	0.01781 to 0.01861
Plateau	-8.277e-005 to 0.0002328	0.0007185 to ???
PercentFast	???	???
KFast	??? to 0.05474	???
PercentSlow	??? to 81.10	(Very wide)
KSlow	0.02762 to ???	???
Kmedium	0.08915 to ???	-0.001610 to ???
Half Life (Slow)	??? to 25.10	???
Half Life (Fast)	12.66 to ???	???
Half Life (Medium)	??? to 7.775	??? to +infinity
Goodness of Fit		
Degrees of Freedom	10153	8121
R squared	0.6276	0.6347
Sum of Squares	0.3425	0.09629
Sy.x	0.005808	0.003443
AICc	-104608	-92183
Normality of Residuals		
Shapiro-Wilk (W)	0.4616	0.8534
P value	<0.0001	<0.0001
Passed normality test (alpha=0.05)?	No	No
P value summary	****	****
Constraints		
PercentFast	0 < PercentFast < 100	0 < PercentFast < 100

PercentSlow	0 < PercentSlow < 100	0 < PercentSlow < 100
KSlow	KSlow > 0	KSlow > 0
Number of points		
# of X values	10160	8128
# Y values analyzed	10160	8128

Supplementary Table 2: Membrane Fragility Assay

Media	MEF +/+	Clone8	Clone9	Clone10	Clone15 (sptbn1 +/+)
**1x**					
One-phase association					
Best-fit values					
Y0	= 1.000	= 1.000	= 1.000	= 1.000	= 1.000
Plateau	1.318	1.061	1.765	1.259	1.277
K	0.7750	Unstable	0.3012	1.705	1.351
Tau	1.290	Unstable	3.320	0.5866	0.7403
Half-time	0.8944	Unstable	2.301	0.4066	0.5131
Span	= 0.3178	= 0.06145	= 0.7651	= 0.2588	= 0.2766
95% CI (profile likelihood)					
Plateau	1.288 to 1.347	1.044 to 1.079	1.705 to 1.825	1.233 to 1.285	1.238 to 1.315
K	0.1751 to ???	(Very wide)	0.1385 to ???	0.2971 to ???	0.1424 to ???
Tau	??? to 5.710	(Very wide)	??? to 7.222	??? to 3.366	??? to 7.023
Half-time	??? to 3.958	(Very wide)	??? to 5.006	??? to 2.333	??? to 4.868
Goodness of Fit					
Degrees of Freedom	544	544	544	544	544
R squared	0.009629	0.0009181	0.01862	0.007769	0.004070
Sum of Squares	65.24	24.38	259.1	50.81	111.4
Sy.x	0.3463	0.2117	0.6902	0.3056	0.4525
Constraints					
Y0	Y0 = 1	Y0 = 1	Y0 = 1	Y0 = 1	Y0 = 1
K	K > 0	K > 0	K > 0	K > 0	K > 0
Number of points					
# of X values	546	546	546	546	546
# Y values analyzed	546	546	546	546	546
**1.5x**					
One-phase association					
Best-fit values					
Y0	= 1.000	= 1.000	= 1.000	= 1.000	= 1.000
Plateau	1.087	-9332	1.212	1.121	1.324

K	Unstable	3.861e-008	1.340	0.5714	0.6155
Tau	Unstable	25899400	0.7461	1.750	1.625
Half-time	Unstable	17952096	0.5172	1.213	1.126
Span	= 0.08661	= -9333	= 0.2120	= 0.1212	= 0.3244
95% CI (profile likelihood)					
Plateau	1.068 to 1.105		1.182 to 1.242	1.094 to 1.149	1.269 to 1.380
K	(Very wide)		0.1658 to ???	0.1066 to ???	0.06366 to ???
Tau	(Very wide)		??? to 6.030	??? to 9.385	??? to 15.71
Half-time	(Very wide)		??? to 4.180	??? to 6.505	??? to 10.89
Goodness of Fit					
Degrees of Freedom	544		544	544	544
R squared	0.001775		0.004018	0.002148	0.003101
Sum of Squares	25.04		66.53	56.34	226.2
Sy.x	0.2145		0.3497	0.3218	0.6449
Constraints					
Y0	Y0 = 1	Y0 = 1	Y0 = 1	Y0 = 1	Y0 = 1
K	K > 0	K > 0	K > 0	K > 0	K > 0
Number of points					
# of X values	546	546	546	546	546
# Y values analyzed	546	546	546	546	546

0.5x

One-phase association					
Best-fit values					
Y0	= 1.000	= 1.000	= 1.000	= 1.000	= 1.000
Plateau	1.196	1.395	1.811	1.822	1.672
K	0.2808	0.2780	0.1600	0.1307	0.1854
Tau	3.561	3.597	6.249	7.652	5.394
Half-time	2.468	2.493	4.332	5.304	3.739
Span	= 0.1957	= 0.3949	= 0.8106	= 0.8225	= 0.6724
95% CI (profile likelihood)					
Plateau	1.166 to 1.226	1.369 to 1.421	1.761 to 1.861	1.791 to 1.855	1.618 to 1.727
K	0.08500 to ???	0.1510 to 0.67[illegible]	0.09785 to 0.2[illegible]	0.09378 to 0.1[illegible]	0.09224 to 0.5169
Tau	??? to 11.76	1.484 to 6.622	3.352 to 10.22	5.262 to 10.66	1.934 to 10.84
Half-time	??? to 8.155	1.029 to 4.590	2.324 to 7.084	3.647 to 7.391	1.341 to 7.515
Goodness of Fit					
Degrees of Freedom	539	539	539	539	539
R squared	0.006473	0.02674	0.04366	0.1130	0.02133
Sum of Squares	65.58	48.82	167.2	66.20	204.6
Sy.x	0.3488	0.3009	0.5570	0.3505	0.6161
Constraints					

Y0	Y0 = 1	Y0 = 1	Y0 = 1	Y0 = 1	Y0 = 1
K	K > 0	K > 0	K > 0	K > 0	K > 0
Number of points					
# of X values	546	546	546	546	546
# Y values analyzed	546	546	546	546	546

Supplementary Table 3: FRAP analysis

Treatment	Actin	βII-spectrin
Untreated		
Mobile Fraction	0.7176 ± 0.12	0.7956 ± 0.07
Half-time Recovery (sec)	11.34 ± 8.56	9.147 ± 4.648
n	25	25
Jasplakinolide		
Mobile Fraction	0.0769 ± 0.22	0.6869 ± 0.14
Half-time Recovery	(1735 ± 7408)	24.53 ± 51.52
n	19	28
Blebbistatin		
Mobile Fraction	0.809 ± 0.14	0.6358 ± 0.08
Half-time Recovery	15.47 ± 6.59	18.93 ± 19.23
n	19	27

Supplementary Table 4: Model Parameters

Parameter	Unit	Value
ξ : drag coefficient	pN s/nm	1.25
Δt : time step length	s	0.002
Spectrin		
$d_{0,S}$: resting length	nm	180
$k_{s,S}$: spring constant	pN/nm	1
F_{th}: force threshold for detachment	pN	0.05
Cable element		
k_c: cable constant	pN/nm	150
Stress fibers		
$k_{s,F}$: spring constant	pN/nm	4
$k_{c,F}$: cable constant	pN/nm	0.222
$d_{0,F}$: resting length	nm	155.8846
t_s: time for active vertical movement of stress fibers	s	300
Focal Adhesion		
v_A: velocity	nm/s	1.0667
Myosin Linkers		
$k_{c,L}$: cable constant	pN/nm	0.25
d_{min}: minimum length	nm	135

d_{max}: maximum length	nm	450
Myosin rods		
$k_{c,M}$: myosin rod cable constant	nm	0.1071
φ_a: myosin rod addition rate	1/s	0.01
φ_r: myosin rod removal rate	1/s	0.0063

Supplementary Table 5

REAGENTS or RESOURCES		
Antibodies	Source	Identifier
Mouse anti-βII-Spectrin	BD Bioscience	BD-612563
Rabbit anti-βII-Spectrin	Abcam	AB-72239
Rabbit anti-αII-Spectrin	Invitrogen	PA5-35383
Mouse anti-βI-Spectrin	NeuroMab	N385/21
Rabbit anti β-Actin	Cell Sign	D6A8
Rabbit anti-Adducin	Abcam	AB51130
Mouse-anti-Tubulin	Sigma	T9026
Mouse anti-Fibronectin	Thermo Fisher	MA5-11981
Mouse anti-Gapdh	Merck	6C5-CB1001
Phalloidin AlexaFluor488	Invitrogen	A12379
Phalloidin AlexaFluor568	Invitrogen	A12380
Rabbit-anti mouse HRP	BioRad	1706516
Mouse-anti rabbit HRP	BioRad	1706515
Donkey anti-mouse AlexaFluor488	Thermo Fischer	A21202
Donkey anti-rabbit AlexaFluor488	Thermo Fischer	A21206
Goat anti-mouse ATTO647N	Sigma	50185
Goat anti-rabbit ATTO647N	Sigma	40839
Goat anti-mouse ATTO594	Sigma	76085
Phalloidin Abberior STAR 635	Merck	30972
Goat anti-mouse CF568	Sigma	SAB4600082
Chicken anti-rabbit CF568	Sigma	SAB4600426
DAPI	Sigma	D9542

RINGER's buffer for Live Microscopy	COMPOSITION
1x (≈300 mOsm)	150 mM NaCl, 1mM MgCl2, 1mM CaCl2, 20 mM Hepes (pH 7.4), 5 mM KCl and 2g/l glucose
0.5x	75 mM NaCl, 1mM MgCl2, 1mM CaCl2, 10 mM Hepes (pH 7.4), 2.5 mM KCl and 1g/l glucose
1.5x	225 mM NaCl, 1mM MgCl2, 1mM CaCl2, 30 mM Hepes (pH 7.4), 7.5 mM KCl and 3g/l glucose

Plasmids	Source	Identifier
GFP-βII-Spectrin	This manuscript	
GFP-Actin	MBI	
RFP-Actin	MBI	
mCherry- βII-Spectrin	This manuscript	
cpst-βII-Spectrin FL	This manuscript	
cpst-βII-Spectrin ΔABD	This manuscript	
RFP-Myosin Light Chain	MBI	

GFP-Myosin IIA	MBI	
TrueGuide Syn sgRNA *sptbn1*	Invitrogen	CRISPR516991_SGM
pSpCas9(BB)-2A-GFP (PX458)	Addgene	48138

Cell lines	Source	
Immortalized MEF (RPTP α +/+ background)	Sheetz' Lab	JCellBiol. 2003 14;161(1):143-53
Immortalized MEF (RPTP α +/+ background, *Sptbn1* -/- clones)	Gauthier' Lab	This paper

Reagents	Source	Identifier
Nitric acid	Sigma	438073
Blebbistatin	Sigma	B0560
Jasplakinolide	Sigma	J4580
Hexamethyldisilazane	Sigma	440191
Sylgard 184 silicone elastomere kit	Dow Corning	1064291
PEG-PLL	Ruixibio	R-PL1226
Silicone membrane	SMI	
Dow Corning® high-vacuum silicone grease	Sigma	Z273554-1EA

Cell culture Reagents	Source	Identifier
DMEM High Glucose	Lonza	BE12-614F
Fetal Bovine Serum	Euroclone	ECS0182L
Trypsin-EDTA	Euroclone	ECB3052D-20
Penicillin Streptomycin	Euroclone	ECB3001L
Fibronectin	Roche	11080938001
Jasplakinolide	Merck	J4580-100UG
Blebbistatin	Sigma	B0560-1MG
Cucurbitacin E	Sigma	SML0577-5MG
Latrunculin A	Sigma	L5163
Cytochalasin B	Sigma	C6762
CK666	Sigma	SML006-5MG
SMIFH2	Sigma	S4826-5MG
Rhodamine-labelled Transferrin	Invitrogen	1865638
CellTox™ Green Cytotoxicity Assay	Promega	G8743
mTESR	Stem Cells	100-0267
Matrigel hESc-qualified	Corning	354277
DMEM-F12	Gibco	31331-093
L-Glutamine	Euroclone	ECB3000D
NEAA	Biowest	X0557-100
N2	Thermo Scientific	21103049
Y27632	AdooQ	A11001
Neurobasal	Thermo Scientific	21103049
B27	Thermo Scientific	17504-044
NT-3	Peprotech	450-03
BDNF	Peprotech	450-02
Laminin	Sigma	L2020
Doxycycline	Sigma	D9891
PLO	Sigma	P3655

ExM Reagents	Brand	

MA-NHS	Sigma	730300
Sodium Acrylate	Sigma	408220
4HT	Sigma	176141
Proteinase K	NEB	P8107S

Microscopes	Brand
TIRF	Leica
SP5 laser scanning confocal	Leica
SP8 laser scanning confocal	Leica
Spinning Disk Confocal Unit	Olympus
Thunder	Leica
Envision XCite	Perkin Elmer

Devices	Source
Aluminum coverslip holder	Nils Gauthier
Micropatterning Quartz Mask	IFOM

Software	Brand
LAS X	Leica
CellSense	Olympus
Prism	Graphpad
Image Lab (5.0)	Biorad
Fiji	NIH
Huygens Professional	SVI
R studio	Cran
Illustrator	Adobe
