# Supplementary material for: Mechanically induced topological transition of spectrin regulates its distribution in the mammalian cell cortex
Source: Nat Commun. 2024 Jul 8;15:5711. doi: 10.1038/s41467-024-49906-6 (PMC11231315; doi:10.1038/s41467-024-49906-6)
Supplement: Supplementary file 3 — Description of Additional Supplementary Information [file 41467_2024_49906_MOESM3_ESM.docx]

**Mechanically induced topological transition of spectrin regulates its distribution in the mammalian cell cortex**

Andrea Ghisleni^1^, Mayte Bonilla-Quintana^2^, Michele Crestani^1&^, Zeno Lavagnino^1^, Camilla Galli^1$^, Padmini Rangamani^2*^, Nils Gauthier^1*^

^1^IFOM ETS, The AIRC Institute of Molecular Oncology, Via Adamello 16, 20139, Milan, Italy

^2^Department of Mechanical and Aerospace Engineering, University of California San Diego, La Jolla CA 92093, USA

^&^Laboratory of Applied Mechanobiology, Department for Health Sciences and Technology, ETH Zürich, CH-8092 Zürich, Switzerland

^$^Humanitas Cardio Center, IRCCS Humanitas Research Hospital, Via Manzoni 56, 20089, Rozzano (Milan), Italy

* corresponding authors: [nils.gauthier@ifom.eu](mailto:nils.gauthier@ifom.eu) ; [prangamani@ucsd.edu](mailto:prangamani@ucsd.edu)

**Inventory of supporting information**

**Supplementary information** Supplementary figures 1-10, Supplementary Tables 1-4 and Resource table (Supplementary Table 5)

**Supplementary Movie 1** Time lapse microscopy of MEF transfected with GFP-βII-spectrin and RFP-Actin, and clusters segmentation

**Supplementary Movie 2** Time lapse TIRF microscopy of MEF transfected with GFP-βII-spectrin and RFP-Actin under jasplakinolide and blebbistatin treatment

**Supplementary Movie 3** Computational modelling of the spectrin network in two initial configurations: compressed and extended

**Supplementary Movie 4** Computational modelling of the spectrin network when elements detachment is OFF and ON

**Supplementary Movie 5** Computational modelling of the spectrin network when stress fibers are hooked by focal adhesions or behave as compressive elements

**Supplementary Movie 6** Time lapse TIRF microscopy of MEF transfected with GFP-βII-spectrin and RFP-MLC, and MLC particle tracking at zones characterized by different GFP-βII-spectrin densities

**Supplementary Movie 7** Computational modelling of the spectrin network compressed by stress fibers, in presence of single and dual dynamic myosin

**Source Data Files**
