# Supplementary material for: Mechanically induced topological transition of spectrin regulates its distribution in the mammalian cell cortex
Source: Nat Commun. 2024 Jul 8;15:5711. doi: 10.1038/s41467-024-49906-6 (PMC11231315; doi:10.1038/s41467-024-49906-6)
Supplement: Supplementary file 11 — Reporting Summary [file 41467_2024_49906_MOESM11_ESM.pdf]

nature portfolio

Corresponding author(s): Nils Gauthier, Padmini Rangamani

Last updated by author(s): 2024/06/12

Reporting Summary

Nature Portfolio wishes to improve the reproducibility of the work that we publish. This form provides structure for consistency and transparency in reporting. For further information on Nature Portfolio policies, see our Editorial Policies and the Editorial Policy Checklist.

Statistics

For all statistical analyses, confirm that the following items are present in the figure legend, table legend, main text, or Methods section.

n/a	Confirmed	
☐	☒	The exact sample size (*n*) for each experimental group/condition, given as a discrete number and unit of measurement
☐	☒	A statement on whether measurements were taken from distinct samples or whether the same sample was measured repeatedly
☐	☒	The statistical test(s) used AND whether they are one- or two-sided<br>*Only common tests should be described solely by name; describe more complex techniques in the Methods section.*
☒	☐	A description of all covariates tested
☐	☒	A description of any assumptions or corrections, such as tests of normality and adjustment for multiple comparisons
☐	☒	A full description of the statistical parameters including central tendency (e.g. means) or other basic estimates (e.g. regression coefficient) AND variation (e.g. standard deviation) or associated estimates of uncertainty (e.g. confidence intervals)
☐	☒	For null hypothesis testing, the test statistic (e.g. *F*, *t*, *r*) with confidence intervals, effect sizes, degrees of freedom and *P* value noted<br>*Give P values as exact values whenever suitable.*
☒	☐	For Bayesian analysis, information on the choice of priors and Markov chain Monte Carlo settings
☒	☐	For hierarchical and complex designs, identification of the appropriate level for tests and full reporting of outcomes
☒	☐	Estimates of effect sizes (e.g. Cohen's *d*, Pearson's *r*), indicating how they were calculated

Our web collection on statistics for biologists contains articles on many of the points above.

Software and code

Policy information about availability of computer code

Data collection	All softwares used to collect data were commercially available and are indicated in Table 4: Resources.<br>LAS x (Leica) and CellSense (Olympus). For western blot the software Image Lab 5.0 (Biorad) was used
Data analysis	Softwares used for data analysis are indicated in Table 4: Resources, and include open source (Fiji, R studio) or licensed softwares (Matlab, Graphpad Prism).

For manuscripts utilizing custom algorithms or software that are central to the research but not yet described in published literature, software must be made available to editors and reviewers. We strongly encourage code deposition in a community repository (e.g. GitHub). See the Nature Portfolio guidelines for submitting code & software for further information.

Data

Policy information about availability of data

All manuscripts must include a data availability statement. This statement should provide the following information, where applicable:

- Accession codes, unique identifiers, or web links for publicly available datasets
- A description of any restrictions on data availability
- For clinical datasets or third party data, please ensure that the statement adheres to our policy

All dataset generated in this study will be made publicly available at the time of acceptance through open access repositories. We have no restrictions to data availability and no clinical or third party datasets are included in this manuscript

Human research participants

Policy information about studies involving human research participants and Sex and Gender in Research.

Reporting on sex and gender	not applicable to the current manuscript
Population characteristics	not applicable to the current manuscript
Recruitment	not applicable to the current manuscript
Ethics oversight	not applicable to the current manuscript

Note that full information on the approval of the study protocol must also be provided in the manuscript.

Field-specific reporting

Please select the one below that is the best fit for your research. If you are not sure, read the appropriate sections before making your selection.

- [x] Life sciences
- [ ] Behavioural & social sciences
- [ ] Ecological, evolutionary & environmental sciences

For a reference copy of the document with all sections, see nature.com/documents/nr-reporting-summary-flat.pdf

Life sciences study design

All studies must disclose on these points even when the disclosure is negative.

Sample size	Sample size was determined based on the experience in previous studies by the authors, in particular Ghisleni, A., et al., Nat Commun, 2020 for the live imaging and FRAP assays, Hastings, R., et al., Cardiov Gen, 2016 for the FRET assay, and other publications that involved live and fixed image analysis
Data exclusions	no data exclusions was performed in the current manuscript
Replication	all the experiments presented were repeated independently at least 3 times or more
Randomization	When relevant, randomization was achieved by shuffling the order of the experimental conditions when performing fluorescence microscopy assays
Blinding	Blinding is not relevant to the present manuscript

Reporting for specific materials, systems and methods

We require information from authors about some types of materials, experimental systems and methods used in many studies. Here, indicate whether each material, system or method listed is relevant to your study. If you are not sure if a list item applies to your research, read the appropriate section before selecting a response.

Materials & experimental systems

n/a	Involved in the study	
[ ]	[x]	Antibodies
[ ]	[x]	Eukaryotic cell lines
[x]	[ ]	Palaeontology and archaeology
[x]	[ ]	Animals and other organisms
[x]	[ ]	Clinical data
[x]	[ ]	Dual use research of concern

Methods

n/a	Involved in the study	
[x]	[ ]	ChIP-seq
[x]	[ ]	Flow cytometry
[x]	[ ]	MRI-based neuroimaging

Antibodies

Antibodies used	all the antibodies used in this study, commercial source and identifier are reported in Table 4: Resources. This table is included in the submitted manuscript
Validation	Validation statements are provided by the distributors

Eukaryotic cell lines

Policy information about cell lines and Sex and Gender in Research

Cell line source(s)	immortalized MEF were obtained from the following study JCellBiol. 2003 14;161(1):143-53. hiPSC-derived cortical neurons were generated by IFOM Cell Culture Facility. Murine erythrocytes were obtained from mice blood, in accordance with the guidelines established in the Principles of Laboratory Animal Care (directive 86/609/EEC) and were approved by the Italian ministry of Health (Project code: 75DA4.N.PGY.)
Authentication	none of the cell line used were authenticated
Mycoplasma contamination	The cell line under investigation in this study have been confirmed negative for mycoplasma contamination
Commonly misidentified lines (See ICLAC register)	n/a
