# Supplementary figures and images for: Mechanically induced topological transition of spectrin regulates its distribution in the mammalian cell cortex
Source: Nat Commun. 2024 Jul 8;15:5711. doi: 10.1038/s41467-024-49906-6 (PMC11231315; doi:10.1038/s41467-024-49906-6)

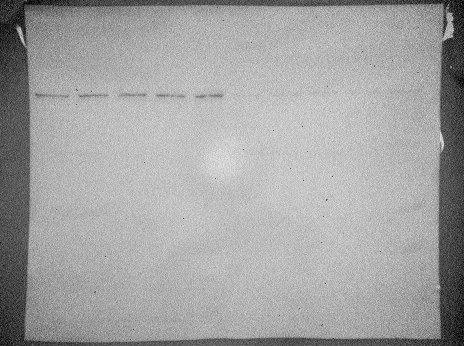

Supplement: Supplementary file 12 — Source data [file 41467_2024_49906_MOESM12_ESM.zip › Source Data Files/Source Data Supplementary/FigureS8/FigureS8b/20210127_rptpdrugtreatment_antisptan.tif]

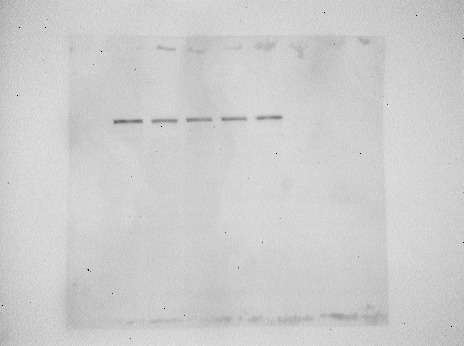

Supplement: Supplementary file 12 — Source data [file 41467_2024_49906_MOESM12_ESM.zip › Source Data Files/Source Data Supplementary/FigureS8/FigureS8b/20210416_rptp_drugtreatments_sptbm_highexp.tif]

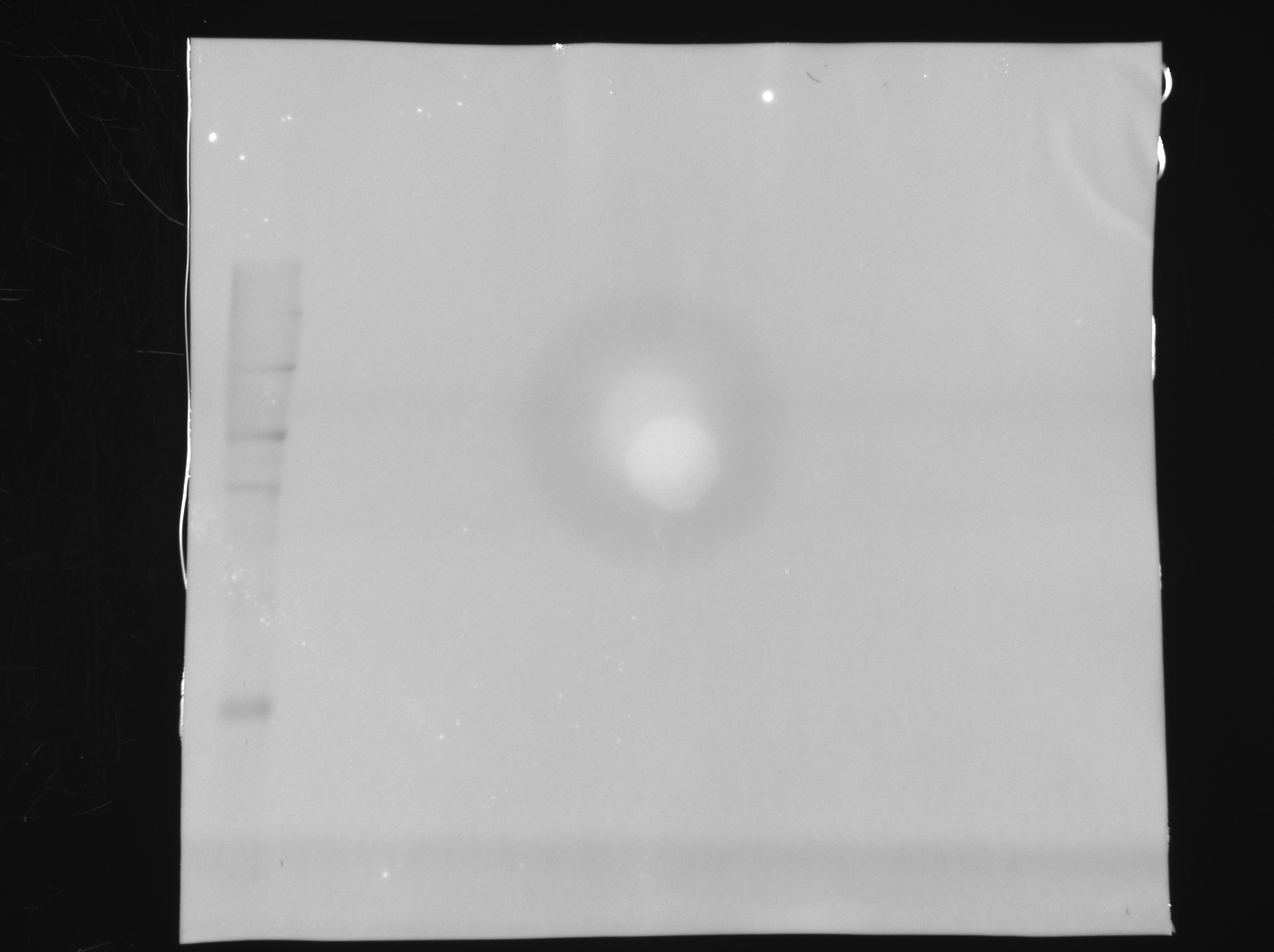

Supplement: Supplementary file 12 — Source data [file 41467_2024_49906_MOESM12_ESM.zip › Source Data Files/Source Data Supplementary/FigureS8/FigureS8b/20210416_rptp_drugtreatments_tubulinMW_lowexp.tif]

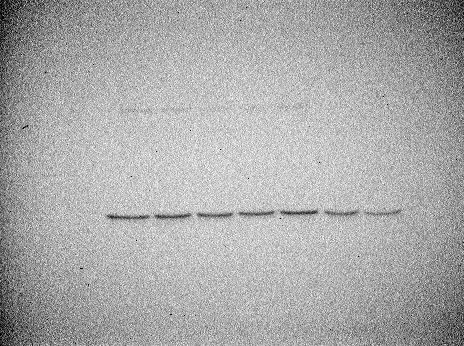

Supplement: Supplementary file 12 — Source data [file 41467_2024_49906_MOESM12_ESM.zip › Source Data Files/Source Data Supplementary/FigureS8/FigureS8b/20210416_rptp_drugtreatments_tubulin_highexp.tif]

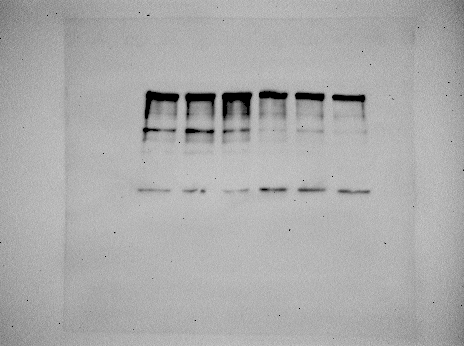

Supplement: Supplementary file 12 — Source data [file 41467_2024_49906_MOESM12_ESM.zip › Source Data Files/Source Data Supplementary/FigureS8/FigureS8c/20211222_rptp_ABD_FL_cpstdrugs_tubulin.tif]

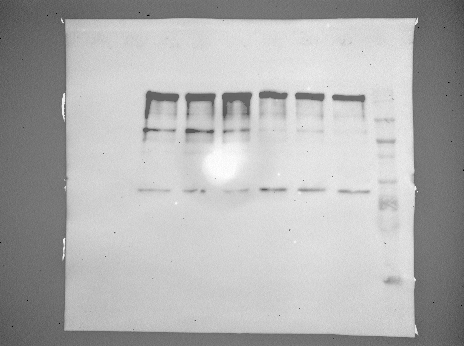

Supplement: Supplementary file 12 — Source data [file 41467_2024_49906_MOESM12_ESM.zip › Source Data Files/Source Data Supplementary/FigureS8/FigureS8c/20211222_rptp_ABD_FL_cpstdrugs_tubulin_MW.tif]

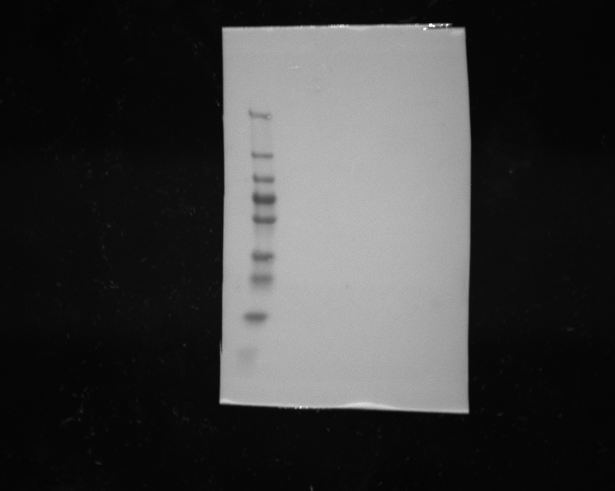

Supplement: Supplementary file 12 — Source data [file 41467_2024_49906_MOESM12_ESM.zip › Source Data Files/Source Data Supplementary/FigureS2/FigureS2a/tubulin/CHEMI_08072023_140155_(Membrane)_raw.tif]

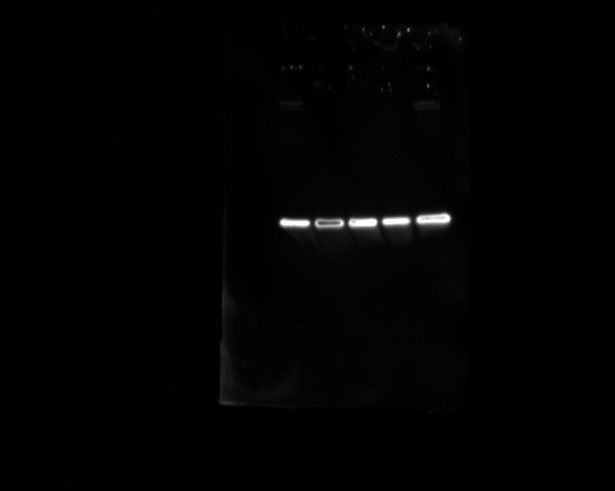

Supplement: Supplementary file 12 — Source data [file 41467_2024_49906_MOESM12_ESM.zip › Source Data Files/Source Data Supplementary/FigureS2/FigureS2a/tubulin/CHEMI_08072023_140155_(Chemi)_raw.tif]

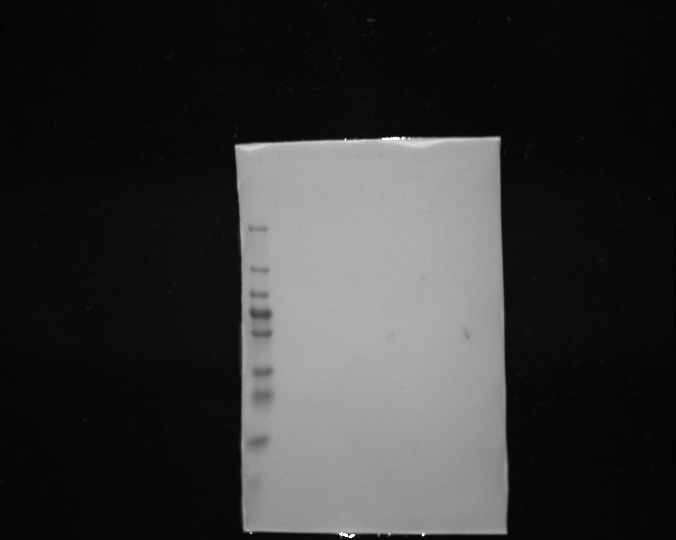

Supplement: Supplementary file 12 — Source data [file 41467_2024_49906_MOESM12_ESM.zip › Source Data Files/Source Data Supplementary/FigureS2/FigureS2a/sptan1/CHEMI_08042023_121354_(Membrane)_raw.tif]

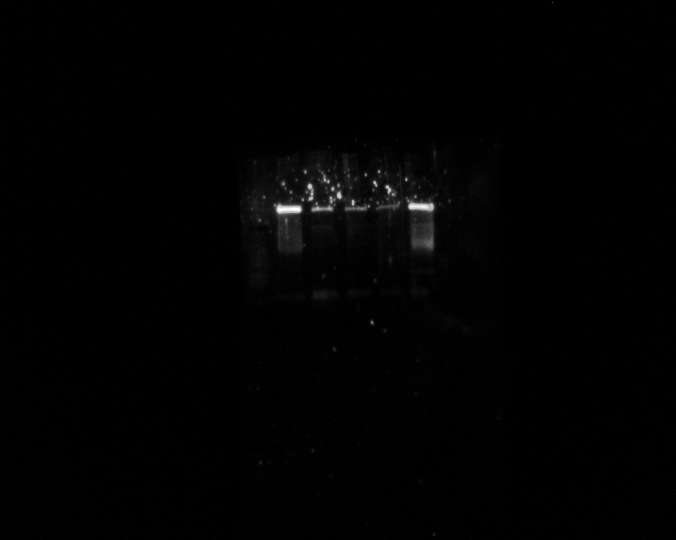

Supplement: Supplementary file 12 — Source data [file 41467_2024_49906_MOESM12_ESM.zip › Source Data Files/Source Data Supplementary/FigureS2/FigureS2a/sptan1/CHEMI_08042023_121354_(Chemi)_raw.tif]

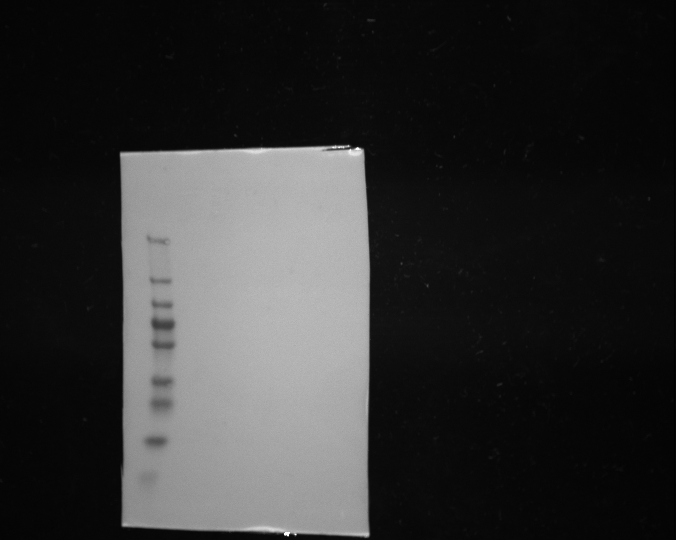

Supplement: Supplementary file 12 — Source data [file 41467_2024_49906_MOESM12_ESM.zip › Source Data Files/Source Data Supplementary/FigureS2/FigureS2a/sptbn1/20230804_wt_8_9_10_15_sptbn1_(Membrane)_raw.tif]

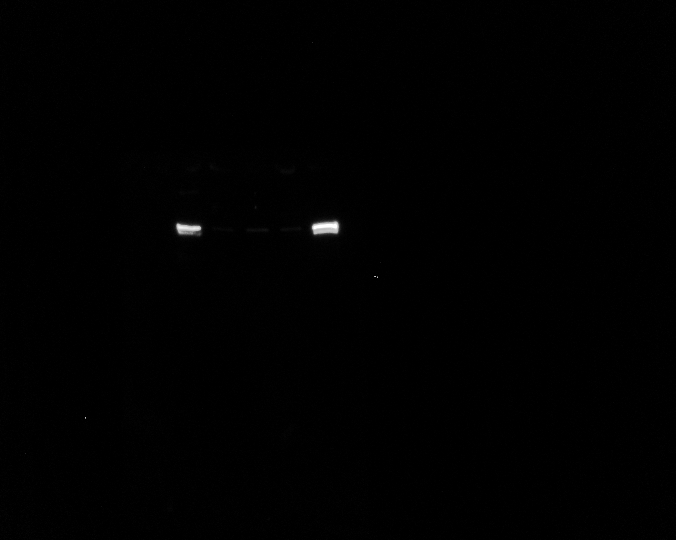

Supplement: Supplementary file 12 — Source data [file 41467_2024_49906_MOESM12_ESM.zip › Source Data Files/Source Data Supplementary/FigureS2/FigureS2a/sptbn1/20230804_wt_8_9_10_15_sptbn1_(Chemi)_raw.tif]

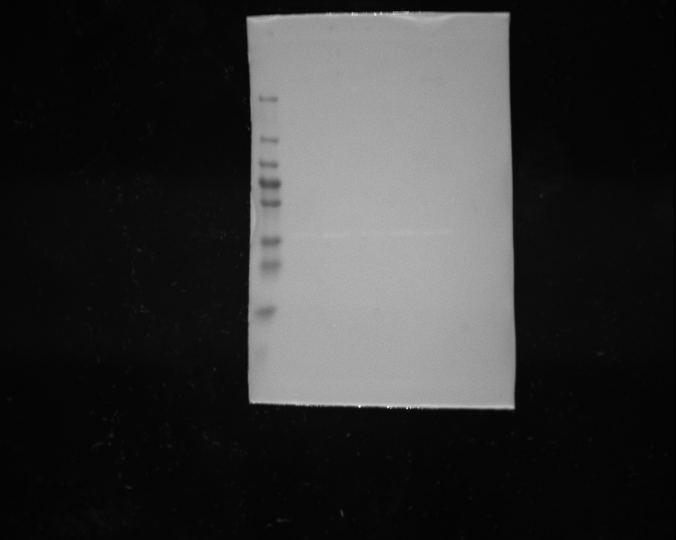

Supplement: Supplementary file 12 — Source data [file 41467_2024_49906_MOESM12_ESM.zip › Source Data Files/Source Data Supplementary/FigureS2/FigureS2a/gapdh/CHEMI_08072023_140610_(Membrane)_raw.tif]

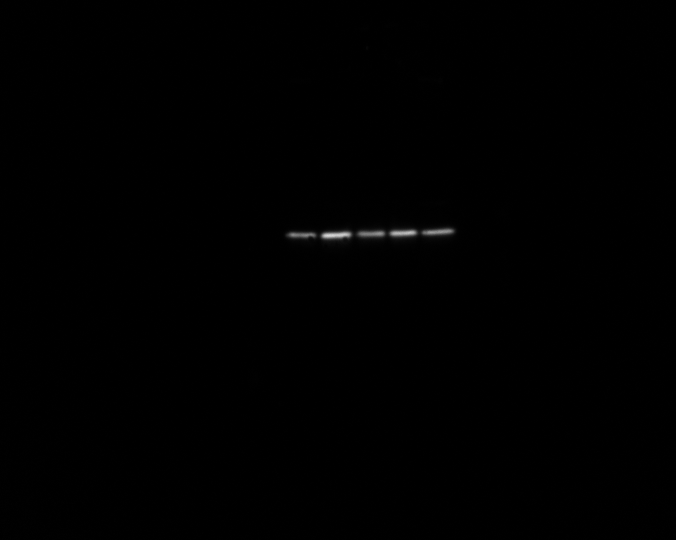

Supplement: Supplementary file 12 — Source data [file 41467_2024_49906_MOESM12_ESM.zip › Source Data Files/Source Data Supplementary/FigureS2/FigureS2a/gapdh/CHEMI_08072023_140610_(Chemi)_raw.tif]
